# Shiga Toxin Is Transported into the Nucleoli of Intestinal Epithelial Cells via a Carrier-Dependent Process

**DOI:** 10.3390/toxins2061318

**Published:** 2010-06-07

**Authors:** Boris Baibakov, Rakhilya Murtazina, Christian Elowsky, Francis M. Giardiello, Olga Kovbasnjuk

**Affiliations:** GI Division, Department of Medicine, Johns Hopkins University School of Medicine, Baltimore, MD, USA; Email: BorisB@intra.niddk.nih.gov (B.B.); rakhilya@hotmail.com (R.M.); celowsky21@yahoo.com (C.E.); fgiardi@jhmi.edu (F.M.G.)

**Keywords:** Shiga toxin, nucleolar trafficking, intestinal epithelial cells

## Abstract

Shiga toxin (Stx) produced by the invasive *Shigella dysenteriae* serotype 1 (*S. dysenteriae*1) causes gastrointestinal and kidney complications. It has been assumed that Stx is released intracellularly after enterocyte invasion by *S. dysenteriae*1. However, there is little information about Stx distribution inside *S. dysenteriae*1-infected enterocytes. Here, we use intestinal epithelial T84 cells to characterize the trafficking of Stx delivered into the cytosol, in ways that mimic aspects of *S. dysenteriae*1 infection. We find that cytoplasmic Stx is transported into nucleoli. Stx nucleolar movement is carrier- and energy-dependent. Stx binding to the nucleoli of normal human enterocytes *in vitro* supports possible roles for nucleolar trafficking in toxin-induced intestinal pathology.

## 1. Introduction

The illnesses caused by the Shiga toxin (Stx)-producing bacteria *Shigella dysenteriae* serotype 1 (*S. dysenteriae*1) can produce fever, fatigue, anorexia, watery and bloody diarrhea, and often progresses to dysentery and the hemolytic uremic syndrome [1,2,3]. Stx-related pathology has been intensively studied, because Shigellosis is a major cause of death of children in the developing world [3,4]. Moreover, *S. dysenteriae*1 strains are often resistant to many drugs, can cause large epidemics and are not prevented or treated by currently-available vaccines [2,3,4].

*S. dysenteriae*1, which is ingested from contaminated food and water or transmitted from infected individuals, causes illness after colonization of the distal small intestine and colon. The bacteria invade the mucosa through M-cells, bypassing the epithelial cell barrier [5]. This microorganism secretes factors such as IpaB, which bind to caspase-1 and initiate phagosome lysis. This phagosome lysis allows bacteria to escape into the cytoplasm. The *S. dysenteriae*1 then multiply and disseminate into the neighboring intestinal epithelial cells (IEC) through the basolateral membrane [5,6]. Severe bloody diarrhea occurs more frequently in persons infected with strains that produce high amounts of Stx, in comparison to those that produce low levels [2,4], suggesting a positive correlation between the amount of toxin and the severity of gastrointestinal complications. 

Stx represents a family of so-called AB_5_ toxins. A pentamer of B subunits is responsible for receptor binding, while a catalytic A subunit possesses *N*-glycosidase activity and inhibits protein synthesis through specific removal of the adenine base at the 28S ribosomal RNA of eukaryotic ribosomes [7,8,9]. 

Our knowledge of the mechanisms of Stx interaction with human enterocytes [1,7,8] is mainly based on studies of the interaction between the purified holotoxin or its recombinant B-subunits (StxB) in IEC models. This approach has several limitations. For instance, the interaction of Stx with its receptor, a glycosphingolipid globotriaosylceramide (Gb3), which has been elucidated in these cell models, probably does not occur in human disease because Gb3 expression in normal enterocytes is undetectable in physiological conditions [10,11,12,13,14]. Moreover, the potential contribution of bacterial infection to the mechanisms of Stx endocytosis and intracellular trafficking has been poorly incorporated in these simplistic *in vitro* IEC cell models. The mechanisms whereby Stx interacts with human enterocytes, the ways in which toxin is distributed and trafficked intracellularly in these cells, and the ways in which Stx mediates changes in cellular signal transduction pathways in the context of *S. dysenteriae*1 infections have not been adequately characterized. 

Because *S. dysenteriae*1 are invasive, Stx is presumably released into the cytoplasm of enterocytes. We now characterize the mechanism for intracellular trafficking of Stx delivered into the cytoplasm of the human intestinal epithelial T84 cell model, as it might occur in *S. dysenteriae*1 infection. We show that Stx, when released into the cytoplasm, is transported into the nucleoli. This nucleolar trafficking of Stx is specific, since neither B-subunit of Cholera toxin (CTB) nor 40 KDa dextran, when delivered into the cytoplasm, enter the nucleoli. Stx nucleolar uptake is not due to diffusion, but rather is an active carrier-dependent process. We discuss the ways in which this newly characterized Stx nucleolar trafficking pathway might contribute to *S. dysenteriae*1-induced intestinal diseases.

## 2. Results and Discussion

### 2.1. Stx delivered into the cytoplasm appears in nucleoli of IEC

**Figure 1:**
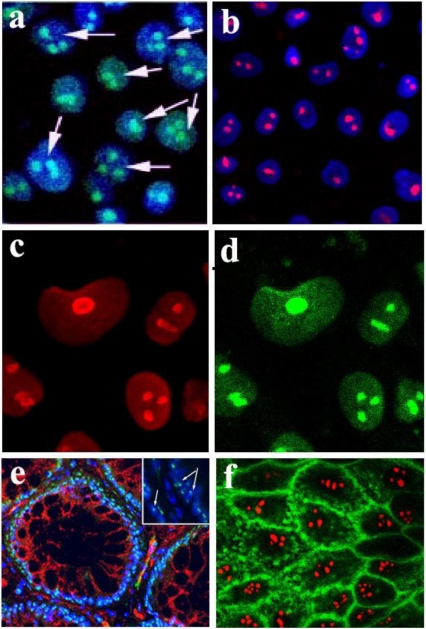
Localization of Stx and StxB to the nucleoli of intestinal epithelial T84 cells and human colonic tissue. (**a**) Stx-Alexa 488 (green) appears in the nucleoli of PM-permeabilized confluent T84 cells; blue: nuclear staining by Hoechst dye; arrows point to the nucleoli filled with Stx-Alexa 488. (**b**) Recombinant StxB-Alexa 568 (red) is delivered from cytoplasm into the nucleoli of PM-permeabilized confluent T84 cells; blue: nuclear staining by Hoechst dye. (**c**) T84 cells immunostained with an antibody against the nucleolar protein nucleolin (red). (**d**) Same cells as in **c** stained with StxB-Alexa 488 (green), confirming StxB nucleolar localization. (**e**) Normal human colonic tissue immunostained with StxB-Alexa 488 (green) indicates the presence of StxB binding sites in nucleoli of crypt epithelial cells; red: β-catenin; blue: Hoechst nuclear stain; arrows in the magnified insert point to the nucleoli inside the nuclei filled with StxB-Alexa 488. (**f**) StxB-Alexa 568 (red) is accumulated in the nucleoli of PM-permeabilized T84 cells, while CTB-fluorescein (green) is present in cytoplasm and membranes but not in nucleoli.

To characterize the distribution of Stx released into the cytoplasm of IEC, as a model for *S. dysenteriae*1 infection, we used a digitonin-based cell membrane permeabilization technique. Digitonin is widely used to deliver viral proteins into the cytosol and has been used to analyze intracellular trafficking of viral proteins [15,16]. As reported, digitonin selectively permeabilizes cellular membranes rich in cholesterol, as is true for the plasma membrane (PM), while intracellular organelle membranes with relatively low cholesterol content, including the nuclear envelope, remain intact [17,18]. Incubation of PM-permeabilized T84 cells with fluorescently-labeled Stx (0.2 µg/mL) led to the accumulation of Stx in nucleoli in ~90% of these cells (Figure 1a).

The B-subunit of Stx has been shown to be necessary and sufficient for Stx retrograde trafficking [7,8,9,19,20]. To test whether StxB is sufficient for toxin movement into subcellular compartments including the nucleolus, PM-permeabilized T84 cells were exposed to recombinant StxB. Fluorescently-labeled StxB (0.5 µg/mL) delivered into the cytoplasm of T84 cells appeared in nucleoli (Figure 1b), indicating that StxB is sufficient for the toxin to enter the nucleoli. Moreover, the pattern of StxB fluorescence in nucleoli was similar to the pattern of staining for the endogenous nucleolar protein nucleolin (Figure 1c, d). Thus, StxB delivered into the cytoplasm accumulates in the nucleoli of T84 cells.

Stx nucleolar accumulation in IEC does not appear to be limited to T84 cells. When we stained normal human colon samples with fluorescently-labeled StxB, nucleoli in both surface (data not shown) and crypt epithelial cells (Figure 1e) were labeled by StxB. Stx-binding sites appear to be present in nucleoli of normal mammalian enterocytes. 

### 2.2. Transport into the nucleoli displays specificity for Stx

Another well-studied member of the AB_5_ toxin family is a cholera toxin (CT). The B-pentamer of CT (CTB) binds to the glycosphingolipid receptor G_M1_ that is expressed at the apical surface of enterocytes. Then the toxin-receptor complex is internalized by retrograde pathways [21]. To test whether CTB can also be transported into the nucleoli upon its cytoplasmic delivery, permeabilized T84 cells were simultaneously exposed at 37 ºC to both fluorescently-labeled StxB and CTB (0.5 µg/mL). CTB appeared mostly in the cytoplasm and at the apical cell surface where it binds to its receptor G_M1_. CTB did not accumulate in nucleoli of T84 cells (Figure 1f). Nucleolar trafficking is thus not a common feature of all AB_5_ toxins, but is rather a characteristic of Stx.

### 2.3. StxB moves into nuclei as a pentamer

Upon production by bacteria, the B-subunits of Stx spontaneously form pentamers in solution. These B-pentamers are the functional units in Stx retrograde trafficking [7,8,9]. In our PM-permeabilized cell model, the StxB is delivered from the cytoplasm into the nucleoli. However, it is conceivable that the StxB pentamer experiences partial degradation by some cytosolic enzyme and that B-subunit fragments might thus contribute to the observed nucleolar toxin accumulation. To determine whether intact StxB pentamers move into the nuclei and accumulate in nucleoli, PM-permeabilized T84 cells were exposed to StxB for 1 h at 37 ºC and then lysed. Total cell lysate was subjected to non-denaturing gel electrophoresis. The appearance of StxB in total cell lysate was detected by Western blot analysis using monoclonal antibody against StxB. Additionally, to explore the possibility that cytoplasmic enzymes might cleave StxB, total cell lysate and nuclear lysate from T84 cells were separately incubated with recombinant StxB, and again subjected to non-denaturing gel electrophoresis. 

**Figure 2:**
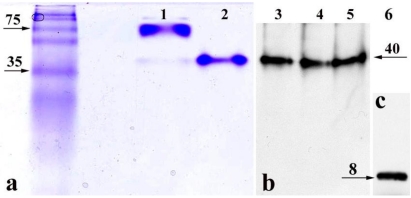
Non-denaturing gels demonstrate that StxB pentamer is present in the nucleoli and cytoplasm of T84 cells. (**a**) Coomassie Blue staining, **lane 1**: Stx holotoxin (10 µg/lane); **lane 2**: recombinant StxB pentamer (5 µg/lane). (**b**) Western blot analysis using mAb against StxB, **lane 3**: StxB from the lysate of permeabilized T84 cells exposed to StxB for 1 h; **lane 4**: StxB after incubation with T84 cell lysate; **lane 5**: StxB after incubation with T84 cell nuclear fraction. (**c**) Recombinant (100 ng/lane) StxB monomer on Western blot following SDS-PAGE. In **lanes 3**–**5**, the molecular weight of StxB is the same as recombinant StxB pentamer in **lane 2**. Images in **b** and **c** were obtained by scanning two membranes simultaneously. Neither Coomassie Blue staining nor Western blot detected any StxB-positive protein bands with molecular weight lower than the StxB pentamer in non-denaturing conditions.

The StxB that accumulated inside the permeabilized T84 cells and StxB after *in vitro* incubation with total cell or nuclear lysate (Figure 2b) appeared on Western blots as a single band of the same molecular weight as purified recombinant StxB identified by Coomassie Blue staining (Figure 2a, line 2). This is smaller than Stx holotoxin band (Figure 2a, line 1). As a control for the antibody sensitivity to StxB monomer, recombinant StxB was subjected to SDS-PAGE under reducing conditions followed by immunoblotting (Figure 2c), which showed that StxB exists as a monomer only. In the cell and nuclear lysates, StxB was similar in size to the recombinant protein on non-denaturing gels (Figure 2a). No bands smaller than the StxB pentamer were detected, indicating that StxB inside the cells is probably not cleaved. These data demonstrate that StxB exists as a pentamer in the cytoplasm and the nuclei of intestinal epithelial cells, and that pentameric StxB is probably the form that enters nucleoli.

### 2.4. StxB does not enter the nucleus by diffusion

Nucleolar protein trafficking includes transport into the nucleus that can occur by two different mechanisms: diffusion or active transport through nuclear pores [22]. The nuclear pore complex (NPC) contains a ~10 nm channel that allows nonselective passive diffusion of small molecules across the nuclear envelope [23]. Small proteins with molecular weights of less than 30–40 kDa can diffuse through the NPC and equilibrate between nucleus and cytoplasm, although larger molecules cannot diffuse across this channel [24]. The molecular weight of Stx holotoxin is ~70 kDa, significantly above this diffusion limit. However, molecular modeling demonstrates that the toxin dimensions are defined by the size of the B-pentamer (molecular weight ~38 kDa) [1,9]. Since 40 kDa molecules can move through the nuclear pore either by a transporter-mediated process or diffusion, we first consider the possibility that StxB movement from the cytosol into the nucleus might occur by diffusion.

To study the mechanism of StxB nuclear transport, we incubated PM-permeabilized T84 cells at 37 °C with both StxB (0.5 µg/mL) and 40 kDa dextran (1 µg/mL), which is transported into the nucleus exclusively by diffusion [25]. We compared the patterns and rates of nuclear accumulation of these two molecules. 

**Figure 3:**
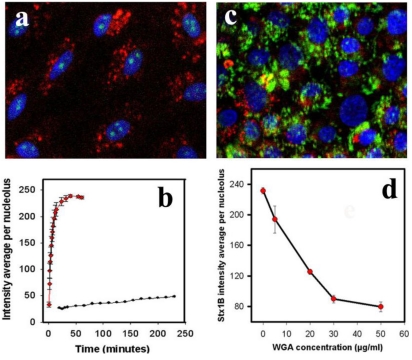
StxB nucleolar trafficking is not due to diffusion through the nuclear pore complex. (**a**) representative confocal image of PM-permeabilized T84 cells incubated with both StxB-Alexa 488 (0.5 µg/mL; green) and 40 kDa dextran conjugated with TMRE (1 µg/mL; red). StxB accumulated in nucleoli, while dextran was present exclusively in the cytoplasm. Blue: Hoechst nuclear labeling; (**b**) The kinetics of StxB nucleolar accumulation (red squares) compared to 40 kDa dextran (black dots) depicted in **a**. The data presented are mean ± SEM of average fluorescence intensity measured in grey levels (g.l.) of 10 nucleoli from 3 different experiments. StxB accumulated significantly faster in the nucleoli than the same molecular weight dextran, achieving a half saturation level in ~6 min, while the amount of dextran did not reach saturation in the nucleoli even after 240 min; (**c**) representative confocal image of T84 cells pretreated for 30 min with 50 µg/mL WGA labeled with fluorescein (green), which prevented transport of StxB-Alexa 568 (red) into nuclei and nucleoli; (**d**) Pretreatment of T84 cells with WGA significantly inhibited StxB nuclear accumulation in a concentration dependent manner. The amount of nucleolar StxB was calculated from confocal images taken 15 min after StxB cytoplasmic delivery. The data presented are mean ± SEM of average fluorescence intensity measured in g.l.

As shown in Figure 3a, after 5 min of incubation, StxB is present in nucleoli, whereas 40 kDa dextran is present only in the cytoplasm (present at undetectable levels in nucleus). 

Quantifying StxB and 40 kDa dextran fluorescence intensities over time in nucleoli (Figure 3b) showed that at a given solution concentration (0.5 µg/mL), the StxB fluorescence intensity in nucleoli reached saturation ~15 min after StxB was added. In contrast, the average nucleolar fluorescence intensity from administration of 40 kDa dextran (solution concentration 1 µg/mL) barely reached a level above background at this time. The lack of dextran detection in nucleoli was not due to lower fluorescence intensity of TMRE or less detection sensitivity; the fluorescence from dextran accumulated in cytoplasm reached saturating levels ~5 min after incubation started (Figure 3a). The large hydrodynamic radius of the 40 kDa dextran molecules is likely to slow diffusion through the nuclear pore, as has been shown previously [26]. Significant amounts of dextran appear in the nucleus only after ~40 min of incubation, indicating that StxB and 40 kDa dextran use different mechanisms of nuclear transport. Thus, StxB does not appear to travel to nucleoli simply by diffusion across nuclear pores. 

To further prove that Stx movement from the cytosol into the nucleus and nucleoli is not via diffusion, T84 cells were treated with the detergent CHAPS, which specifically permeabilizes the nuclear membrane [27,28] and enables molecules to diffuse freely between cytoplasm and nucleoplasm. Accumulation within the nucleus and nucleolus under these conditions can only occur through binding to nuclear or nucleolar components. StxB accumulated in the nucleoli of CHAPS-permeabilized T84 cells in ways similar to the accumulation into cells permeabilized by digitonin treatment. CHAPS did not cause back-diffusion of StxB into the cytoplasm (data not shown). These results add to evidence that diffusion is not responsible for appearance of StxB in nucleus and nucleolus.

The transport of proteins and RNAs through the gated channels in the NPC is a highly selective and signal-mediated process [22,29]. Wheat germ agglutinin (WGA) can bind to *O*-linked glycoproteins of the NPC [30] and inhibit translocation of macromolecules through the nuclear pore without altering free diffusion [31,32]. Pretreatment of T84 cells with WGA for 30 min (Figure 3c) significantly inhibited StxB nucleolar accumulation. 50 µg/mL WGA virtually abolished StxB nuclear transport (Figure 3d), compared to control (Figure 1b). However, 50 µg/mL WGA did not prevent nuclear accumulation of 10 kDa dextran (Supplemental Figure 1). Taken together, these data provide additional evidence that StxB is transferred across nuclear pores by carrier-dependent processes and not by diffusion. 

### 2.5. StxB-subunit nucleolar uptake is carrier dependent

**Figure 4:**
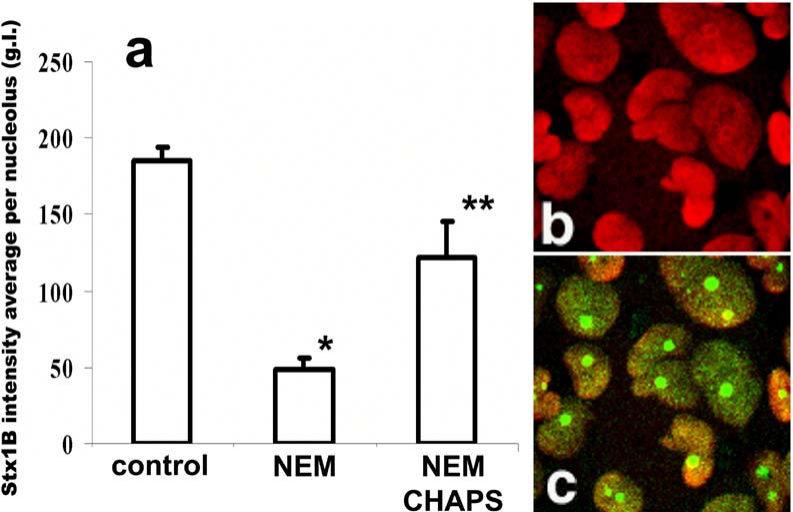
StxB nucleolar trafficking is nuclear localization signal-dependent, but is transcription independent. (**a**) NEM pretreatment (1 mM for 30 min) significantly inhibits StxB nucleolar accumulation compared to levels found in control non-treated cells (*p < 0.05, n = 265 nucleoli). CHAPS permeabilization of NEM-treated cells yielded levels of StxB nucleolar accumulation that were significantly greater than those of cells treated with NEM alone, but less than those of control cells that received neither treatment (** significant changes compared to control and to NEM treatment, p < 0.05, n = 106 nucleoli). The amount of StxB in nucleoli was calculated from 8-bit confocal fluorescence images as described in methods and expressed in g.l. (**b**) Representative immunofluorescence image of T84 cells pretreated with 5 µg/mL ActD, which did not retain nucleolin (red by Alexa 568 secondary antibody) in nucleoli. (**c**) The same cells were still able to transport StxB-Alexa 488 (green) into the nucleoli.

Transport of many proteins into the nucleus is directed by short amino acid sequences known as nuclear localization signals (NLS), which specifically interact with the NPC. Most well-characterized NLS contain a short stretch of basic residues, often flanked by a proline or glycine. Such a prototypical NLS for the SV40 large T-antigen is necessary and sufficient to direct the T antigen from the cytoplasm to the nucleus [33]. However, comparison of the StxB sequence with all known NLS sequences [34] using the BLAST database did not identify any area with a high homology. Because StxB does not have an identified NLS, and because StxB does not diffuse through the nuclear pore (Figure 3), we hypothesized that some protein that has a NLS and can shuttle between the cytoplasm and nucleus might bind StxB in the cytoplasm and carry it into the nucleus and nucleoli. It has been shown [35,36] that nuclear import of many NLS-bearing proteins requires cytosolic factors that are sensitive to alkylation by *N*-ethyl-maleimide (NEM). Thus, we examined the effect of NEM on StxB nucleolar uptake. Pretreatment of permeabilized T84 cells with 1 mM NEM for 10 min decreased the amount of StxB in nucleoli by almost 75% of control values (Figure 4a). These data are consistent with the idea that a NLS-bearing protein might be involved in StxB nuclear/nucleolar trafficking. 

To test whether NEM treatment blocks further StxB binding by modifying the presumed nucleolar StxB receptor, we permeabilized the NEM-pretreated cells with CHAPS and quantified the relative amount of nucleolar Stx1B. The CHAPS treatment partially reversed NEM inhibition of nucleolar StxB accumulation (Figure 4a). StxB nucleolar binding sites are thus rather not inactivated by NEM. During these NEM plus CHAPS experiments, StxB nucleolar intensity did not reached the levels achieved under control conditions (Figure 4a). Carrier-dependent StxB transport from cytoplasm into nucleoli under control conditions is thus likely to be faster than CHAPS-enabled toxin diffusion through the nuclear envelope in NEM-treated cells.

### 2.6. StxB nucleolar accumulation is independent of transcription

Mammalian nucleoli are large (up to 5–10 µm) structures that disassemble in late prophase and reform in telophase in response to transcription of ribosomal DNA [37]. There are three structurally distinguishable nucleolar constituents: fibrillar centers, dense fibrillar components, and granular components, as observed by electron microscopy [38,39]. Transcription likely occurs near the border of the fibrillar center and dense fibrillar components. Accumulation of several proteins in nucleoli, including accumulation of rRNA binding proteins, is transcription-dependent. We thus determined whether StxB nucleolar import was modified by actinomycin D (ActD) treatments that block transcription [40]. T84 cells were incubated for 3 h with either 0.05 or 5 µg/mL ActD in order to inhibit the activity of either RNA polymerase I alone or both RNA polymerase I and II, respectively. The effectiveness of ActD treatment was assessed by dramatic treatment-induced redistribution of the endogenous nucleolar protein nucleolin [41], which became excluded from nucleoli as predicted (Figure 4b). However, neither low nor high doses of ActD prevented StxB movement into the nucleoli (Figure 4c). Quantification of the StxB average fluorescence intensity reflects StxB nucleolar quantities, and did not change after ActD treatment in comparison to control treatment, indicating that StxB nucleolar accumulation is independent of DNA transcription.

### 2.7. StxB is actively transported across the nuclear membrane

Crossing of the NPC is generally an energy- and temperature-dependent process [42,43,44]. To test whether nuclear transport of StxB is temperature dependent, T84 cells where chilled to 4 ºC for 1 h in cold serum free media prior to the exposure to StxB for 15 min. 

**Figure 5:**
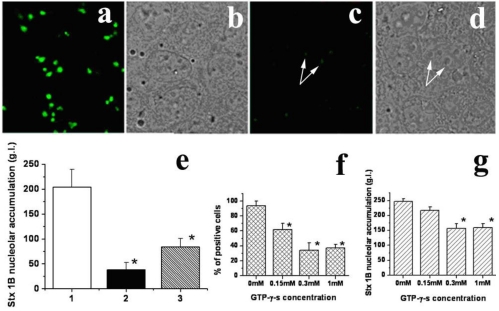
StxB nucleolar transport is temperature and energy-dependent. (**a**) An example of fluorescent StxB (green) accumulated in the nucleoli of permeabilized T84 cells after 15 min of incubation with StxB at 37 ºC. (**b**) The corresponding bright field image of **a**; (**c**) Cells incubated with StxB (green) for 15 min at 4 ºC. (**d**) The corresponding bright field image. White arrows point to nucleoli. (**e**) The relative amount of StxB in the nucleoli of T84 cells calculated from fluorescence images at 37 ºC (**1**), at 4 ºC (**2**) and after ATP depletion at 37 ºC (**3**). Low temperature and ATP depletion significantly decrease the StxB nucleolar amount compared to control cells at 37 ºC (p < 0.05, n = 60 nucleoli for each group). (**f**) GTP-γ-s treatment significantly decreases % of cells with nucleolar StxB in a dose-dependent manner compared to non-treated control cells (*p < 0.05, n = 311 cells for each condition from 10 fields in 3 different preparations). (**g**) GTP-γ-s treatment (0.3 mM) significantly decreases the amount of nucleolar StxB compared to non-treated control cells (* p < 0.05, n = 178 cells from 10 fields in 3 different preparations).

Low temperature significantly inhibited nucleolar transport of StxB (Figure 5c–e) compared to levels observed in control cells incubated at 37 ºC (Figure 5a and b). The StxB nucleolar intensity decreased from 204 ± 36 g.l. in control to 38 ± 7 g.l. at 4 ºC (Figure 5e). Moreover, StxB fluorescence levels fell below the limit of detection in ~40% of cells at 4 ºC. Since passive diffusion is proportional to the absolute temperature, a drop from 37 ºC to 4 ºC would have been expected to exert a much smaller effect [45]. These results provide additional support for the idea that import of StxB does not occur by passive diffusion. 

Nuclear import often involves interaction between cargo and the members of the importin family of carrier molecules, which contains more than 20 human isoforms [46]. This import requires energy. In many cases, at least part of the energy requirement is linked to the small GTPase Ran, which catalyzes the release of cargo from the importin complex inside the nucleus [47,48]. Incubation of T84 cells at 37 ºC with GTP-γ-s, a nonhydrolyzable GTP analog, for 1 h prior to StxB, significantly and dose-dependently reduced both the number of cells able to transport StxB into nucleoli and the amount of StxB in nucleoli. The percent of cells able to transport StxB into nucleoli significantly decreased (p < 0.05) from 94 ± 6% in control to 34 ± 10% in the presence of 300 µM GTP-γ-s (Figure 5f). The amount of nucleolar StxB (Figure 5g) also significantly decreased (p < 0.05) in the presence of 300 µM GTP-γ-s (156 ± 26 g.l.) compared to that in control (247 ± 19 g.l). 

It has recently been shown that ATP depletion causes an inhibition of Ran-dependent nuclear protein transport due to dissociation of ATP-dependent Ran-GDP complexes [49,50]. Upon ATP depletion, by 2 h combined pretreatment of T84 cells at 37 ºC with 24 µM rotenone and 15 mM deoxyglucose [41], the relative amount of StxB in the nucleoli was significantly different (p < 0.05) in control (204 ± 36 g.l.) to in ATP-depleted cells (84 ± 27 g.l.) (Figure 5e), These data show that Stx translocation across the nuclear membrane is an active, energy-dependent process.

The distribution of Stx inside the intestinal epithelial cells from patients infected with invasive *S. dysenteriae*1 has not been characterized. Also, while the role of Stx in intestinal epithelial pathology due to Shigellosis is assumed, the direct impact of Stx itself on intestinal epithelial pathology has not been determined. Here, using a T84 cell model, we define the intracellular trafficking and distribution of Stx delivered into the cytoplasm of IEC as it potentially occurs in *S. dysenteriae*1 infection. Our data show that a cytoplasmic pool of Stx can be transported into the nucleoli of IEC and that StxB is sufficient for this trafficking. This process is efficient; virtually 100% of PM-permeabilized T84 cells exposed to Stx or StxB displayed toxin in their nucleoli. 

Our conclusion that nucleolar uptake of StxB does not occur by diffusion but rather via a carrier-dependent mechanism is based on several lines of experimental evidence, including: (1) A well-characterized nuclear protein import model based on PM-permeabilization by digitonin allows the maintenance of the nuclear membranes in a state competent for signal-dependent translocation through the nuclear pore [17]. Thus, the nuclear and consequently nucleolar appearance of Stx was not due solely to lysis of the nuclear envelope by detergent. (2) StxB nucleolar transport was significantly faster than that of diffusion of similarly-sized 40 kDa dextran [25]; (3) a lectin WGA, which inhibits active nuclear transport but not diffusion, by direct binding to acetylglucosamine-bearing nucleoporins [30], significantly inhibited StxB nucleolar accumulation in a dose-dependent manner; (4) StxB nucleolar accumulation is temperature and energy-dependent, being inhibited by low temperature, ATP depletion and GTP substitution. Taken together, these data demonstrate that StxB is actively translocated across the NPC to enter the nucleus.

Nucleolar accumulation of Stx is specific, because double-labeling of T84 cells with CTB and StxB at 37 ºC showed that, even after 5 h of toxin exposure, only StxB was in the nucleoli, while CTB was present in the cytoplasm and in the membrane structures in the same cells. 

The nuclear import of large molecules is an energy-dependent process mediated by transport factors, many of which belong to the family of importins. Importins bind to cargo proteins in the cytoplasm, aid their translocation through NPCs, and then release them in the nucleus. However, to be recognized by these transport factors, the cargo proteins must contain one or more NLS sequences to direct them into nucleus. About 10 different NLS have been identified, including the classical PKKKPKV sequence from SV40 T-antigen and XPR repeats in Herpes virus type I US11 protein [37]. The StxB amino acid sequence did not have any known NLS. This allowed us to hypothesize that in the cytoplasm, StxB may bind to another protein that bears a NLS and carries StxB through the NPC. Our experiments using NEM, which inhibits nuclear import of NLS-bearing proteins [35,36] demonstrate that StxB nucleolar trafficking significantly decreased in a manner consistent with the involvement of a NLS-bearing protein-carrier.

Many nucleolar proteins, which are involved in ribosomal biogenesis, are able to shuttle between cytoplasm and nucleus. There are often several NLS on one such molecule. Moreover, to accumulate in the nucleoli, such proteins also need so-called nucleolar retention signals [50,51]. However, no common amino acid sequence that targets all nucleolar proteins to nucleoli has been identified. Rather, individual sequences on each molecule serve this role, as in the case of several viral proteins [52]. Binding to some nucleolar protein with a NLS might explain both StxB translocation across the nuclear envelope and its nucleolar retention. This hypothesis is supported by our experimental observation that StxB binds to nucleoli of human enterocytes as detected by immunofluorescence. This data also supports our hypothesis that Stx is transported from the cytoplasm to the nucleoli *in vivo* in the course of *S. dysenteriae*1–induced intestinal disease.

Recent observations in monocyte-derived cells show that StxB can co-localize with the nucleolar marker nucleolphosmin/B23 [53] and can be co-precipitated with B23 [53,54]. However, our preliminary data do not support such a direct interaction between StxB and B23 in IEC. We believe that the Stx nucleolar carrier has not been identified. 

Stx nuclear/nucleolar appearance has been reported previously. Trafficking of Stx holotoxin or StxB in human astrocytoma and ovarian carcinoma cells was identified either to the ER/nuclear envelope or the Golgi/ER, depending on the Gb3 fatty acid isoform present [55]. Nuclear targeting appeared preferentially with Gb_3_ containing shorter chain fatty acids. The authors proposed that the fatty acid chain—dependent traffic of Gb_3_ from the cell surface to the ER/nuclear membrane provides a specific signal transduction pathway for Stx/StxB. In contrast, our data gained in permeabilized T84 cells and also in immunostained human colonic tissue sections showed that the presence of Gb3 and the formation of toxin-Gb_3_ complexes are not necessary for StxB nucleolar localization. Examination of Stx distribution in samples of intestinal tissue from patient infected with *S. dysenteriae*1 is necessary to determine whether Stx trafficking from cytoplasm into the nucleoli takes place in human disease. If Stx appears in the nucleoli of enterocytes due to *S. dysenteriae*1 infection, the role of the nucleolar pool of toxin in disease should be sought with vigor. 

## 3. Conclusions

Here we have characterized a newly-recognized Stx pathway from the cytoplasm to nucleoli of IEC. We also showed that nucleoli of human enterocytes have Stx binding sites, indicating that this newly-described Stx trafficking pathway might be relevant for *S. dysenteriae*1-induced intestinal pathology. Stx nucleolar transport is an active and a carrier-dependent process and is specific for Stx compared to other AB_5_ toxins. 

## 4. Materials and Methods

### 4.1. Materials

Purified Stx and recombinant StxB (GRASP Center, Tufts-New England Medical Center, Boston, MA), were labeled with either Alexa Fluor-488, or Alexa Fluor-568 reactive fluorescent dyes following the manufacturer’s protocol (Invitogen, Molecular Probes, Eugene, OR). Fluorescein conjugated wheat germ agglutinin (WGA), tetramethylrhodamine (TMRE) labeled 40 kDa and 10 kDa dextran, propidium iodide, recombinant Annexin V conjugated with Alexa 488 fluorescent dye, and Alexa 488 or 568 conjugated secondary antibodies were from Invitrogen. Purified cholera toxin B-subunit (CTB) conjugated with fluorescein was from List Biological Laboratories, Inc. (Campbell, CA). Digitonin, NEM, ActD, rotenone, TMRE, deoxyglucose, CHAPS and monoclonal antibody against ß-catenin were obtained from Sigma. Polyclonal anti- nucleolin antibody was a generous gift from Joseph Gall (Carnegie Institution of Washington, Baltimore, MD). Monoclonal CD77 antibody against Gb3 was from Seikagaku America (Falmouth, MA). Monoclonal antibody against StxB was a generous gift from Cheleste Thorpe (Tufts-New England Medical Center, Boston, MA).

### 4.2. Cell culture

Since normal human enterocytes lack Gb_3_, we chose human Cl^-^-secreting colon carcinoma T84 cells as a model to study the interaction of Stx with IEC because this cell line is considered Gb_3_–free [12,13,14,56]. The T84 cells were maintained in Dulbecco’s modified Eagle’s medium (DMEM) supplemented with 25 mM NaHCO_3_, 10 mM HEPES, 50 IU/mL penicillin, 50 μg/mL streptomycin, and 10% fetal bovine serum in a 5% CO_2_ incubator at 37 ºC. For experiments, the cells were grown on plastic Petri dishes or glass coverslips and were used 1–5 days after reaching confluence.

For permeabilization, cells were treated with 30 µg/mL digitonin for ~ 3 min at 37 ºC in serum free media containing 250 mM sucrose as it has been described in detail [26]. With these conditions, the cells were permeabilized without significant cell damage as checked by visible changes in cell shape, propidium iodide labeling and DNA-laddering assays. 

### 4.3. Cell lysate preparation and protein electrophoresis

For total lysate preparation, cells were washed 3 times with ice-cold PBS and lysed in 1 mL of buffer containing 60 mM HEPES-NaOH, pH 7.4, 150 mM NaCl, 3 mM KCl, 5 mM EDTA, 3 mM EGTA, 1mM Na orthovanadate, 50 mM NaF, 1% Triton X-100 and protease cocktail inhibitor (#P8340; Sigma) by sonication 3–4 times for 20 s each. Insoluble cell debris was removed by centrifugation for 20 min at 12,000 x *g* and the supernatant was used as total cell lysate. 

Large-scale preparation of nuclear extracts from T84 cells was performed as described previously [57]. Nuclear pellets were washed twice with PBS and resuspended in buffer A (50 mM HEPES, pH 7.4, 50 mM KCl, 0.1 mM EDTA, 1 mM DDT, 10% glycerol and protease cocktail inhibitors (Sigma)) plus 300 mM (NH_4_)_2_SO_4_. The resuspended nuclei were rocked for 30 min at 4 ºC followed by centrifugation at 350,000 x *g* for 10 min. 

Both total cell lysates and nuclear extracts were immediately incubated with 0.5 µg/mL StxB by rocking for 1 h at room temperature or overnight at 4 ºC and then were analyzed using Native (non-denaturing) PAGE (11%) according to Laemmli [58]. After electrophoresis, proteins were visualized with Coomassie Blue or transferred to nitrocellulose membrane for Western blot analysis with an antibody against StxB.

### 4.4. Immunofluorescence microscopy

*Cell model*: In experiments measuring Stx or StxB accumulation in nucleoli, control or pretreated monolayers were permeabilized for 3 min, washed with serum-containing media to remove digitonin, and incubated with fluorescently-labeled Stx (0.2 µg/mL) or StxB (0.5 µg/mL) at 37 ºC for 15 minutes, after which free fluorescent toxin was washed away by serum-containing media. Cell monolayers were examined by confocal imaging followed by quantitation of toxin nucleolar fluorescence intensity. 

In immunofluorescence experiments, after StxB treatment (0.5 µg/mL), cells were fixed for 10 min in 3% paraformaldehyde, permeabilized with 0.1% CHAPS, treated with a mixture of 2% bovine serum albumin and 15% fetal calf serum to prevent non-specific binding, and incubated for 1 h at room temperature with primary Ab followed by 1 h incubation with fluorescently-labeled secondary Ab. Control and treated cells for each experiment were from the same passage and had equal cell density. 

To quantify the relative amount of fluorescent toxin that was transported into the nucleoli, 8-bit confocal images of the cells were generated using a Zeiss 510/META confocal microscope, as we described in detail [56]. Optical sections from 15 different randomly chosen areas with multiple cells in each control (n = 3) and experimental monolayer (n = 3 for each type of treatment) were collected. Images were inclusively thresholded to eliminate background and ensure no pixels with intensity higher than 255 grey levels (g.l.) were included in the analysis. The average fluorescence intensity in g.l. that corresponds to the amount of fluorescent StxB for each nucleolus in each image was measured (MetaMorph, Universal Imaging, West Chester, PA). The average amount of StxB per nuclei in control monolayers was calculated as mean g.l. ± S.E.M and set at 100% ± S.E.M. The amount of nucleolar toxin in experimental monolayers was calculated as percentage relative to the average amount of toxin in controls. 

In experiments measuring StxB and 40 kDa dextran nucleolar accumulation over time, T84 cells were mounted in a temperature controlled 37 ºC perfusion chamber, as described previously [59]. The monolayers were perfused for 3 min with serum free solution containing digitonin plus StxB-Alexa 488 and 40 kDa dextran-TMRE. Then cells were perfused with complete growth media containing serum. A single confocal optical section through the middle of the majority of cells in the field of view was taken for red and green emission channels simultaneously each 30 sec starting 2 min before digitonin perfusion and continuing for the next 12 min. After that time (to avoid possible bleaching), images from the same optical section were taken every 2 min until the end of the experiment. The relative amounts of StxB and 40 kDa dextran in nucleoli for each time point were calculated from 8-bit images.

*Human normal colonic tissue:* Frozen 20 µm thick samples of surgically removed, normal human colonic tissue were obtained from the Johns Hopkins Colorectal Cancer Registry with JHM-IRB1 granted exemption. Tissue samples were fixed, permeabilized with 0.1% saponin or 0.1% CHAPS and immunostained with fluorescently conjugated StxB and mAb against ß-catenin. Images were obtained using a Zeiss 510/META confocal microscope, as described above.

### 4.5. Statistics

Data presented are mean ± standard error of the mean (S.E.M.). Statistical significance was determined using the Student’s *t* test, and p values ≤ 0.05 were considered statistically significant.

## Figures and Tables

**Appendix 1:**
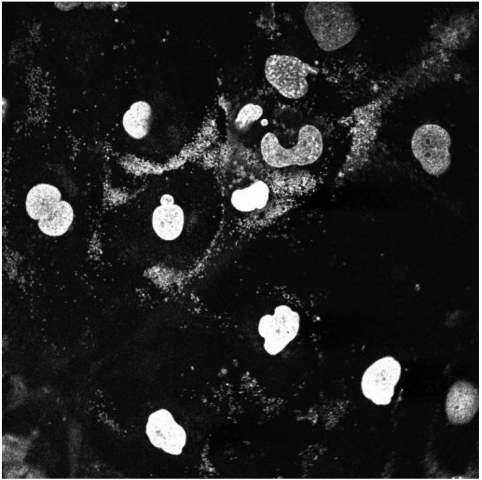
WGA does not inhibit nuclear transport of 10 kDa dextran. Confocal image of T84 cells pretreated for 30 min with 50 µg/mL WGA (not conjugated to a fluorescent dye) and exposed for 5 min to 1 µg/mL 10 kDa dextran-TMRE. Dextran is present in nuclei and in cytoplasm of T84 cells.
